# Spontaneous Activity Characteristics of 3D “Optonets”

**DOI:** 10.3389/fnins.2016.00602

**Published:** 2017-01-09

**Authors:** Anat Marom, Erez Shor, Shulamit Levenberg, Shy Shoham

**Affiliations:** Department of Biomedical Engineering, Technion – Israel Institute of TechnologyHaifa, Israel

**Keywords:** 3D cultures, Neural activity, Neural development, calcium imaging

## Abstract

Sporadic spontaneous network activity emerges during early central nervous system (CNS) development and, as the number of neuronal connections rises, the maturing network displays diverse and complex activity, including various types of synchronized patterns. These activity patterns have major implications on both basic research and the study of neurological disorders, and their interplay with network morphology tightly correlates with developmental events such as neuronal differentiation, migration and establishment of neurotransmitter phenotypes. Although 2D neural cultures models have provided important insights into network activity patterns, these cultures fail to mimic the complex 3D architecture of natural CNS neural networks and its consequences on connectivity and activity. A 3D *in-vitro* model mimicking early network development while enabling cellular-resolution observations, could thus significantly advance our understanding of the activity characteristics in the developing CNS. Here, we longitudinally studied the spontaneous activity patterns of developing 3D *in-vitro* neural network “optonets,” an optically-accessible bioengineered CNS model with multiple cortex-like characteristics. Optonet activity was observed using the genetically encodable calcium indicator GCaMP6m and a 3D imaging solution based on a standard epi-fluorescence microscope equipped with a piezo-electric z-stage and image processing-based deconvolution. Our results show that activity patterns become more complex as the network matures, gradually exhibiting longer-duration events. This report characterizes the patterns over time, and discusses how environmental changes affect the activity patterns. The relatively high degree of similarity between the network's spontaneously generated activity patterns and the reported characteristics of *in-vivo* activity, suggests that this is a compelling model system for brain-in-a chip research.

## Introduction

During the early stages of central nervous system development, neuronal connections are formed. This process is accompanied by spontaneous neuronal activity (Feller, 1999; Blankenship and Feller, 2010), which further stimulate neural connectivity and axonal branching (Katz and Shatz, 1996; Ruthazer and Cline, 2004; Uesaka et al., 2006). Spontaneous activity patterns of this sort have been characterized in 2D neural cell cultures using various methods, in studies which investigated the activity at the cellular level (using intracellular electrodes) and as a population (using extracellular electrodes; Marom and Shahaf, 2002; Wagenaar et al., 2005; Shein et al., 2008). High-density 2D cultures of dissociated cortical cells tend to exhibit spontaneous activity patterns that develop in a stereotyped manner. They begin as uncorrelated sporadic action potentials toward the end of the first week in culture, followed by single-cell activity that combines sporadic and clustered action potentials (Kamioka et al., 1996) and which later develops into synchronized bursting of the network. Bursts occur several times per minute, with durations of 0.5–2 s. In young cultures, bursts tend to be global, but, with age, are replaced by more spatially localized bursts. At early stages of network development, i.e., 9–12 days *in vitro*, the networks exhibit synchronized regular bursting. By 22–33 days, more complicated and diverse patterns appear and the activity becomes synchronized, non-periodic, and clustered (Habets et al., 1987; Maeda et al., 1995). This activity pattern persists for more than 2 months, and is thus considered to be the mature state of the neural network (Marom and Shahaf, 2002).

Neural network formation and neuronal activity have also been studied in three dimensional (3D) neuronal cultures (Edelman and Keefer, 2005; Baker and Chen, 2012), in which neurons are embedded in a 3D scaffold, which enables higher and more complex connectivity (Lai et al., 2012; Ulloa Severino et al., 2016). 3D cultures relevant for activity probing include networks embedded in a extracellular matrix (ECM)-like hydrogel scaffold (Irons et al., 2008; Iwashita et al., 2014) and 3D neuronal cultures devoid of ECM (Bosi et al., 2015), which can be probed using 3D electrode arrays (Musick et al., 2009) or optical (Pautot et al., 2008) approaches. Optical imaging methods, particularly calcium imaging (Cossart et al., 2005; Shein et al., 2008; Herzog et al., 2011; Dana et al., 2014), support neuronal population activity recording at cellular resolution, and avoid the spike sorting problem associated with extracellular electrodes. The optical alternative can also be used to show correlations between neuron activity and morphology (Cossart et al., 2005) and is particularly well suited for distributed 3D populations (Edelman and Keefer, 2005; Irons et al., 2008; Baker and Chen, 2012; Lai et al., 2012; Dana et al., 2014; Bosi et al., 2015; Ulloa Severino et al., 2016). Nevertheless, to the best of our knowledge, no study has thus far characterized the development of spontaneous activity patterns of a large neuronal population in 3D tissue-like cultures embedded in an ECM-like hydrogel scaffold.

In this study, we optically imaged and characterized the activity patterns of developing 3D Matrigel-embedded embryonic (E18) cortical rat neural networks at a single-cell resolution. The 3D transparent neural “optonets” were probed across all different stages of network development, using the genetically encoded calcium indicator (GECI) GCaMP6m (Chen et al., 2013). We describe how complex spontaneous activity patterns develop over time and their modulation by environmental parameters.

## Materials and methods

### Cell culture

Neural cells were extracted by dicing (using a scalpel) cortical tissue from day 18 (E18) embryonic rats in cold PBS solution with 20 mM glucose, mechanically dissociating neural cells, by forcing a few times through a pipette, and filtering the suspension using a 70 μm cell strainer (Biologix). Cells were embedded into a scaffold (see Section Matrigel construct preparation) and cultures were grown in 1.8 mL culture medium composed of Minimal Essential Medium (MEM, without phenol red, Sigma), supplemented with 17 mM glucose, 100 μl/ml NU-serum (BD Biosciences), 30 mg/ml L-glutamine (Sigma), 1:500 B-27 supplement (Gibco), 50 ng/ml nerve growth factor (NGF, Alomone labs), 10 ng/ml brain-derived neurotrophic factor (BDNF, R&D systems), 25 μg/ml insulin (Sigma), and 2 μg/ml gentamicin, while maintaining the cultures floating throughout the culture period (CO_2_ perfusion, 37°C). Half of the volume of the culture medium was replaced twice a week.

### Matrigel construct preparation

Neural cells (1.2 × 10^6^ were suspended in 30 μL Matrigel (growth factor-reduced, without phenol red, BD Biosciences). The matrigel was cooled to 4°C, to its liquid form to allow suspension of the cells. Following incubation at 37°C, the polymerization of Matrigel proteins led to encapsulation of the cells and enabled the formation of a tissue-like 3D construct. The constructs were then placed in culture medium and incubated in a 35 mm Petri dish (CO_2_ perfusion, 37°C).

### Stainings

For whole-gel differential immunostaining, cultures were fixated for 2 h in 4% paraformaldehyde (PFA) solution in PBS, and then permeabilized and blocked with 0.3–1% Triton and 4% fetal bovine serum (FBS) solution in PBS (4 h). The cultures were then washed with PBS, and incubated (overnight, 4°C) with the following primary antibodies: Mouse-Anti beta III tubulin (1:400, Promega), a neuronal cell marker, and Rabbit-Anti S100 (1:200, Sigma), a marker of glial cells. Cultures were then washed with PBS and exposed (4 h to overnight) to CY3-conjugated goat-anti-mouse IgG (1:100, Jackson), Dylight 488-conjugated goat-anti-rabbit IgG (1:100, Jackson), and DAPI (6 nM, Sigma), designed to stain the nuclei of all cells and incubated for at least 4 h, before being washed again with PBS. Cells were imaged using a confocal microscope (Zeiss, LSM 700).

### Calcium imaging

**Figure 1:**
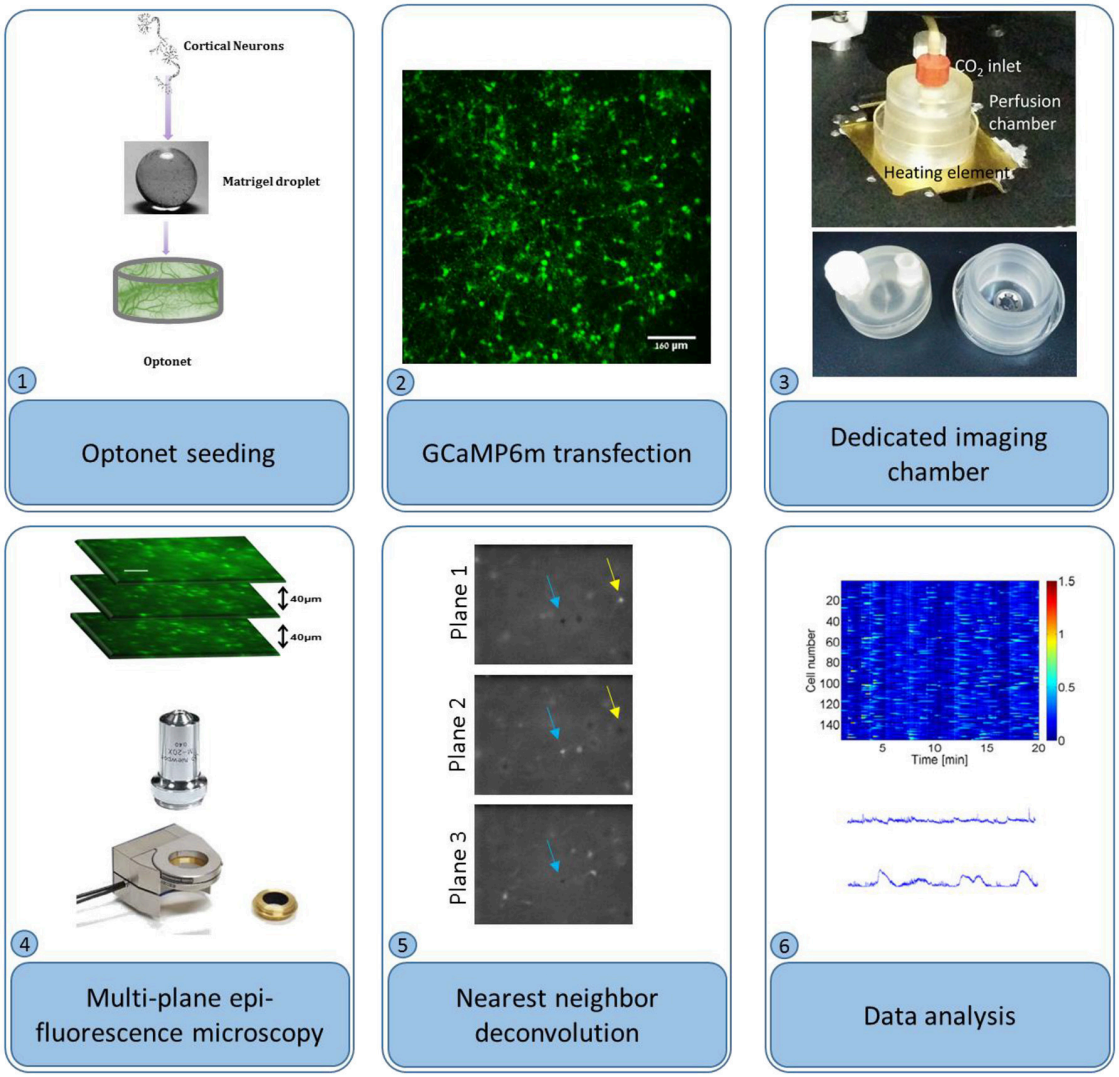
**Optonet preparation and activity probing**. (1) Cortical neurons are encapsulated in a matrigel droplet. (2) The matrigel embedded with neurons was cultured in a bio-reactor and transfected with GCaMP6m AAV. (3) To probe activity, the droplet was maintained in a temperature controlled perfusion chamber. (4) An epi-fluorescence microscope, with a piezo-controlled z stage, was used to image sections. (5) Nearest neighbor deconvolution filtered out cross-talk from neighboring layers. (6) ΔF/F was calculated per neuron and analyzed.

Cultures were transfected with adeno-associated virus (AAV) 2.1, encoding for the genetic calcium indicator GCaMP6m (Grewe et al., 2010) with medium response kinetics, under the synapsin promoter (Penn Vector Core), to be selectively expressed in neurons. The vector was introduced to the entire cell population, at concentrations ranging from 6.69^9^to 22.39^9^ GC/ml. The selected vector concentration depended on the desired imaging time point; to gain sufficient expression for calcium imaging at the first days of the culture *in vitro* higher vector concentrations were required in comparison to imaging at later days, when high concentrations resulted in undesired expression of the indicator. *In vitro* calcium imaging was performed between days 5 and 29 in culture, using a fluorescence microscope (Nikon) equipped with a piezoelectric device (piezosystem jena) capable of moving the objective lens to multiple focal planes. Three planes, separated by 40 μm depth intervals were captured at 4 frames per second each to allow acquisition of each frame (and consequently each neuron) at 4 Hz, in-line with the GCaMP6m decay time (Chen et al., 2013). During the imaging sessions, the cultures were maintained in an incubation chamber (34–37°C), with CO_2_ perfusion under sterile conditions, thus allowing multiple imaging sessions of each culture (Figure 1).

### Neuronal activity analysis

Multiple planes were acquired using an epi-fluorescence microscope with a piezo actuator-controlled objective, which was repeatedly cycled across several positions. Video sequences were separated into planes, and subsequently the cross-talk between planes was reduced using nearest neighbor deconvolution: intra-plane cross-talk was estimated by measuring the dimensions of a small object in a neighboring plane and approximated using a Gaussian PSF. Adjacent planes were convolved with this Gaussian and subtracted from the analyzed plane. (Figure 1) Next, the temporal fluorescence signals were normalized according to ΔF/F(t) = [F(t)-F_0_(t)]/[F_0_(t)], where F_0_ is each cell's 5th percentile fluorescence, calculated using a 25-s moving window to remove slow baseline variations. An activity event was detected when the fluorescence F(t) crossed 50% of the maximum ΔF/F threshold and a burst was detected when 50% or more of the cells were active at a given time point.

### Statistical methods

All error bars represent standard errors. The 5th percentile fluorescence intensity calculated over a moving window of 100 samples, was used as the neuronal activity baseline. Calculations were performed using MALTAB (Mathworks). All results shown are mean ± SEM.

## Results

### Optonets develop spontaneous neuronal activity patterns that change over time

**Figure 2:**
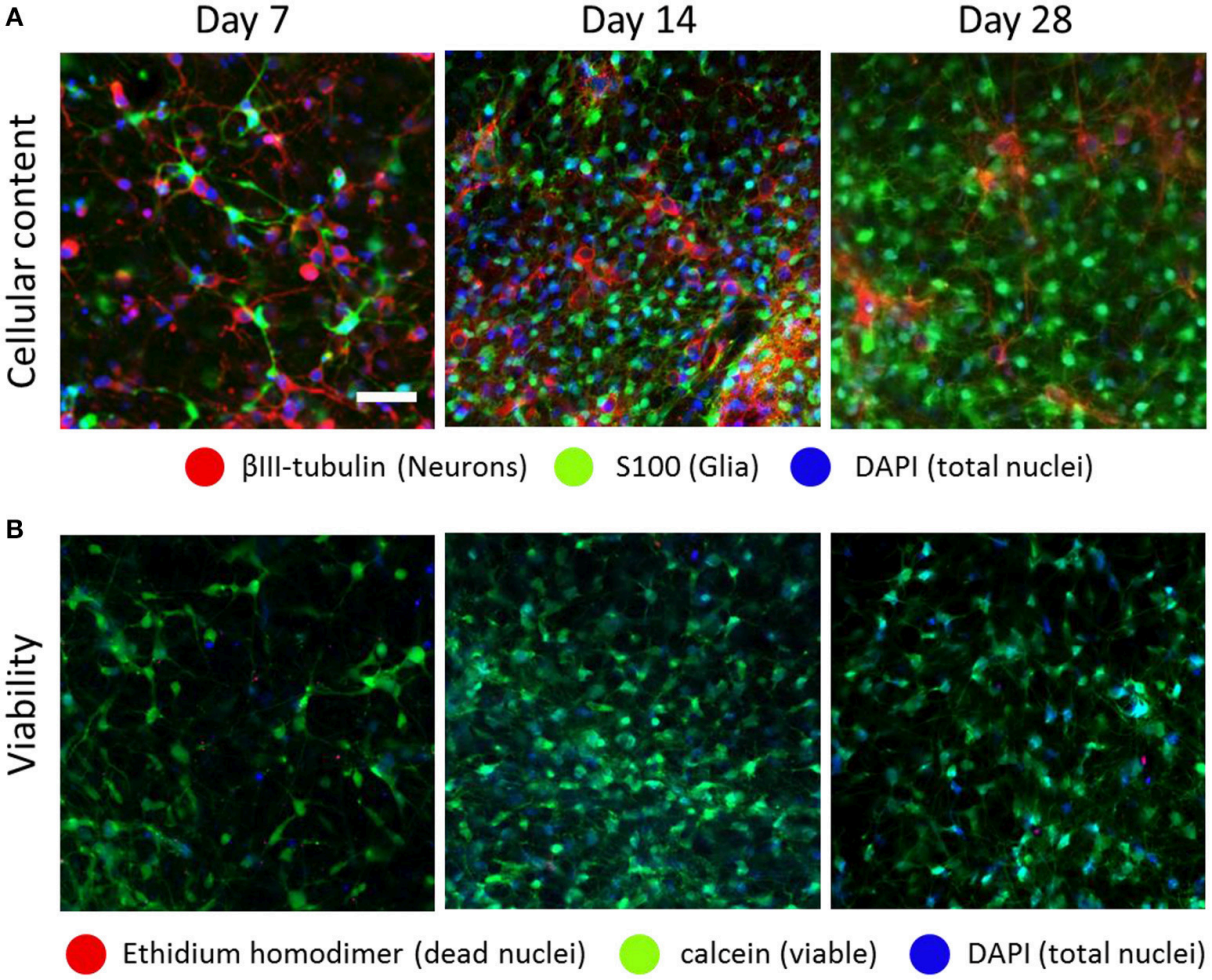
**Optonet development over time**. Confocal images of embryonic (E18) cortical cells embedded in a Matrigel scaffold, which formed a spontaneous network composed of both neurons and glia, at ratios that changed over time **(A)**. The optonets maintained high viability rates during the timeframe in which the experiments were conducted **(B)**. Scale bar- 50 μm.

**Figure 3:**
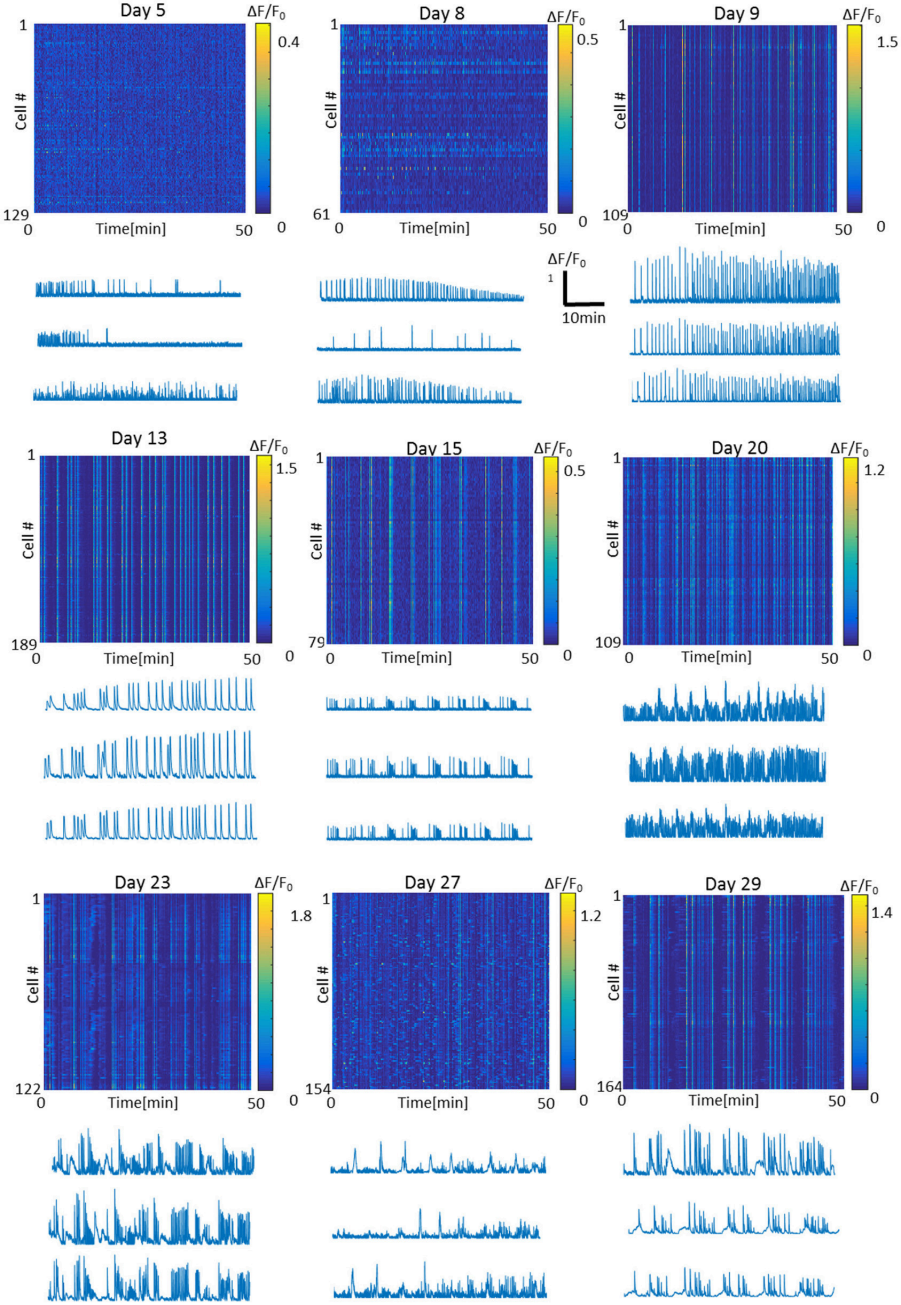
**Representative fluorescence intensity images and traces of the optonet on different days of culture ***in vitro*****. Each line in the intensity image represents one cell in the 3D network. Bursts appear as straight vertical lines in the intensity image. Traces scale: x- Time = 10 min, y- ΔF/F = 1.

To characterize optonet neuronal activity, volumetric activity movies (3 planes, 80 μm stack), from which ΔF/F signal traces were calculated for each cell (Figure 1), were captured from E18 cortical neuron-embedded Matrigel scaffolds over a 24-day period. The optonet cultures were transfected with a GCaMP6m AAV virus driven by a synapsin promoter to express the probe only in neurons and 5 days after transfection they expressed sufficient amounts of the indicator to allow imaging. During maturation, rapid formation of complex neuronal projections was noted between day 7 and day 14. Morphology stabilized after the first 14–21 days and cells remained viable (Figure 2). The cultures demonstrated diverse activity during network development that included sporadic, wave and bursting activity patterns. A collection of representative patterns is provided in Figure 3.

**Figure 4:**
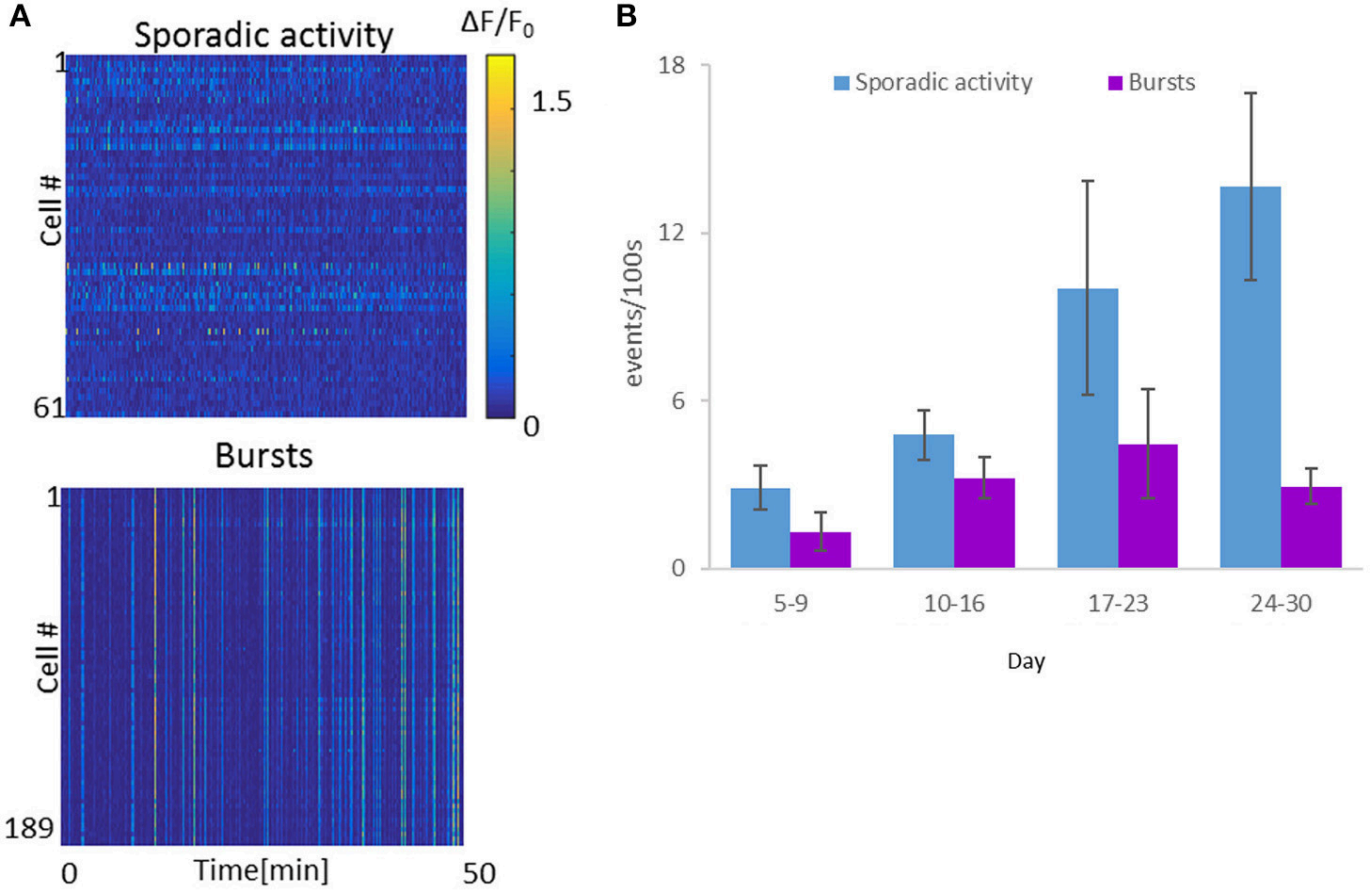
**Spontaneous activity levels of the Optonet**. **(A)** Representative neuronal GCaMP6m activity recordings of sporadic activity (top) and bursts (bottom). Each line represents activity of one cell over 50 min. **(B)** Cell-averaged event rate per 100 s for each sample recorded (1 h recording for each sample), over the *in vitro* culture period. Error bars represent the standard error of the mean (SEM), *n* = 4–8 samples for each group.

To quantify the activity per cell, ΔF/F activity traces were analyzed, with network bursts defined as events in which ≥50% of the cells exhibited ΔF/F > 10%, while all other activity was defined as sporadic (Figure 4A). We calculated event rates of sporadic activity per cell and bursting activity. Throughout the course of the experiment, sporadic activity levels increased as a function of optonet maturity, with average sporadic activity per cell increasing from 2.9 ± 0.8 events per 100 s during days 5–9, to 4.8 ± 0.9 events/100 s on days 10–16. On days 17–23, the average activity level was 10.0 ± 3.8 events/100 s and between days 24 and 30, was as high as 13.7 ± 3.4 events/100 s (in each time range, 494–699 cells were imaged in 4–8 cultures, mean: 4.8 cultures). Burst rates peaked at around day 20, and then declined: In parallel, network burst events during days 5–9 occurred at a rate of 1.3 event per 100 s (6 cultures, total of 699 cells). The rate of burst events further rose between days 10 and 16, averaging 3.2 events/100 s (4 cultures, 628 cells), and peaked at day 17 to day 23, with a mean rate of 4.5 bursts/100 s (5 cultures, 447 cells). Between day 24 and 30, burst rates then declined to 2.9 events/100 s (4 cultures, 610 cells) (Figure 4B).

### Synchronous activity increases, then declines during the maturation of the optonet

**Figure 5:**
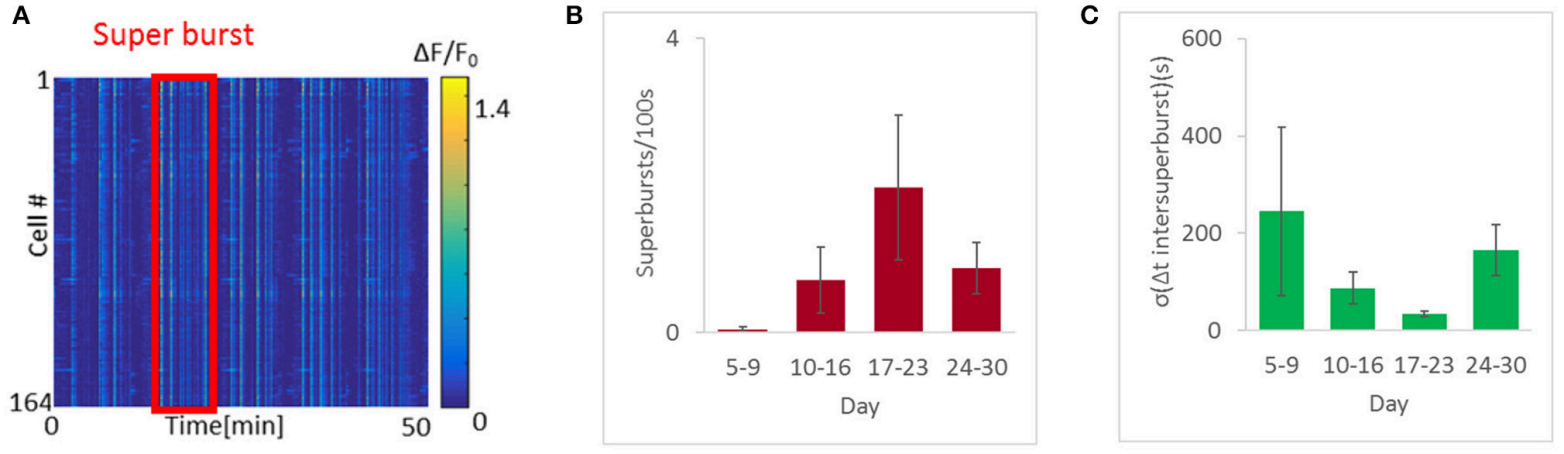
**Highly regular super-synchronous optonet activity. (A)** Sample recording of a super-burst. **(B)** Abundance of super-bursts with developmental age. **(C)** Quantification of super-burst regularity. Error bars represent the standard error of the mean (SEM), *n* = 4–8 samples for each group.

A closer look at the appearance of these bursts revealed another interesting phenomenon. At early stages we observed isolated bursts. Thereafter, the bursts began to cluster into “apparent” long bursts appearing within a short time interval, followed by a pause, a phenomenon termed “superbursts” (Wagenaar et al., 2006; Figure 5A). These superbursts were generally more abundant as burst rates rose, and were most prevalent between days 17 and 23, when a mean 2 superbursts/100 s (6 cultures, 699 cells measured) were recorded. In contrast, fewer than 1 superburst/100 s was observed on days 5–16 and days 24–30 (10 cultures, 1075 cells 4 cultures and 610 cells, respectively). Moreover, the frequency of super-burst firing became more constant during the period associated with the highest firing rate (Figures 5B,C), with an average inter-burst interval of 32 ± 14 s. In contrast, between day 10 and day 16 the interval was 84 ± 70 s, and at days 24–30, the mean interval was 163 ±102 s.

### Activity episodes become longer and more complex with network maturation

**Figure 6:**
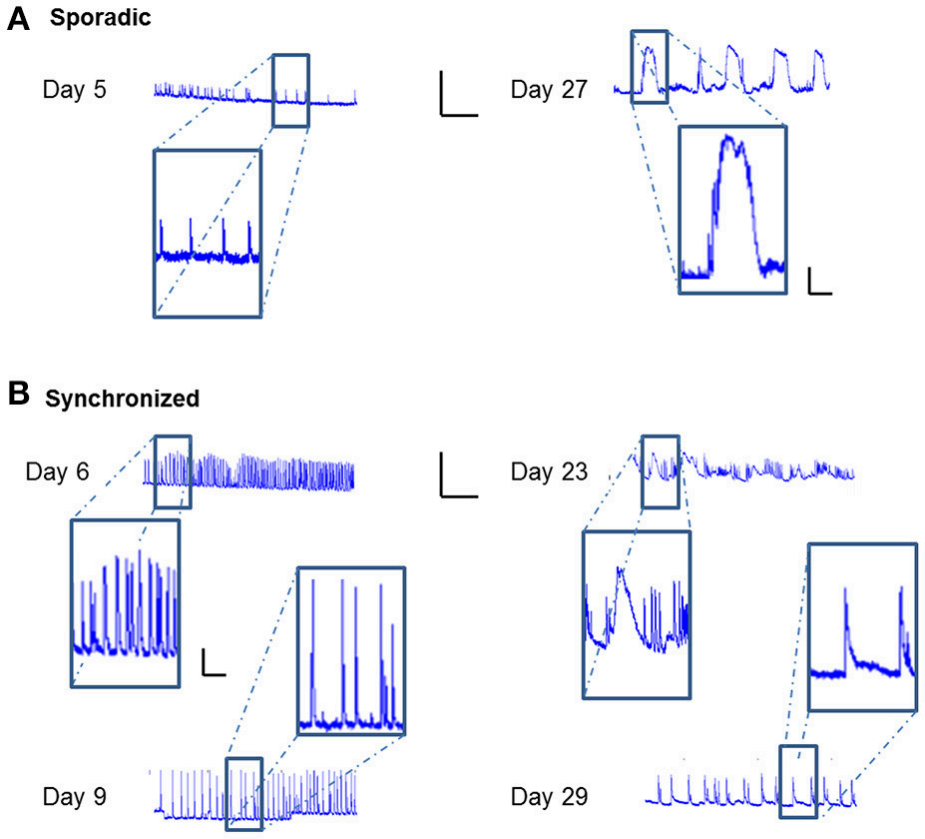
**Temporal prolongation of activity-related events**. ΔF/F traces over time of different cell cultures. **(A)** Traces of sporadic activity-related events (magnified in insets) recorded on Days 8 and 27. **(B)** Traces of synchronized network activity events (magnified in insets) recorded on Days 6, 9, 23, and 29. Traces scale bars - X axis: ΔF/F- 0.5, Y axis: Time- 5 min; Inset scales 0.1, 1 min.

Careful analysis of the fluorescence traces revealed that both sporadic (Figure 6A) and synchronized (Figure 6B) network events of early-stage cultures tended to exhibit a fast rise and decline of fluorescence, unlike mature cultures that exhibited slower fluorescence transitions for both sporadic (Figure 6A) and synchronized (Figure 6B) events. In the former period (days 3–9), the transition period was within 0.5–1 s, consistent with GCaMP6m response of a single action potential. With age, cultures tended to exhibit more complex and diverse activity patterns, as seen by the traces of the changes in fluorescence activity (Figure 6), which presented as smears in the activity maps (Figure 3 days 23, 27, 29), lasting between 10 and 60 s.

### Temperature strongly modulates the network activity characteristics

**Figure 7:**
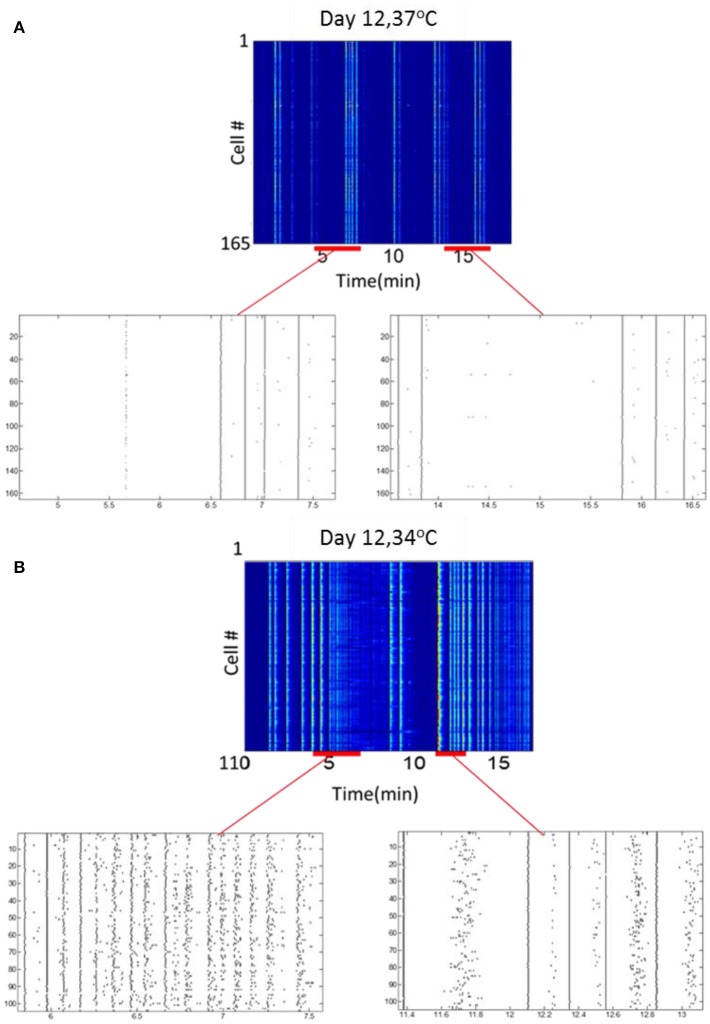
**Temperature modulates neuronal activity. (A)** Activity map and rasters at 37°C. **(B)** Activity map and rasters of the same culture samples, at 34°C.

To examine the effect of thermal environmental variations on optonet activity, the steady-state activity profiles recorded when the temperature of the chamber holding the optonets was physiological (37°C) was compared to those collected in reduced (34°C) temperatures. Our main observation is that the activity patterns forming in the lower temperature were more diverse or less regular and bursatile. Measurements of activity at 36°C was similar to the activity observed at 37°C (data not shown). At 37°C, network bursts with a typical duration of 0.5–1 s were observed at an average frequency of 3.1 ± 0.2 bursts/100 s (165 cells). Sporadic activity was also present but at low rates. The same culture maintained at 34°C (*n* = 110 cells) exhibited more diverse or less regular and bursatile activity. Short network bursts were still present at a similar frequency (3.9 ± 0.4 bursts /100 s), and strong wave-like activity was present, where cells were firing sequentially rather than in complete synchrony. The overall activity rate was higher when the temperature was lower (Figure 7).

## Discussion

In this study, we presented a detailed characterization of the diverse spontaneous activity patterns observed in a 3D-cultured cortical neuronal population model during its development. The cultures were grown in Matrigel scaffolds, where their activity was optically recorded with single cell resolution from as early as day 5, using virally mediated expression of the GECI GCaMP6m. Matrigel was selected as the preferred scaffold for culturing and characterizing optonets (Marom, 2014) after testing several other candidates for their ability to support neural cell growth. The superior ability to support neural network development and formation of a functional 3D network might be rooted in its resemblance to natural brain ECM, its natural origin and its low stiffness (approximately 450 Pa, same order of magnitude as the embryonic brain Iwashita et al., 2014). Up to day 8, the activity was mostly sporadic and unsynchronized, but then switched to bursting activity patterns observed across all cultures starting at day 9. The bursting activity peaked between days 17 and 23, after which, it declined, while sporadic activity continued to rise and became increasingly irregular. The synchronous activity not only peaked during this late-stage period, but also manifested a regular, oscillator-like firing frequency that vanished as the culture further matured.

The recorded developmental profile demonstrated some similarities to those reported for 2D cultures. (1) Both culture types present early onset of sporadic activity [dominant up to day 7 in 2D cortical cultures, (Hinard et al., 2012) vs. day 8 in our data], and subsequently of synchronous activity bursts (day ≤ 6 for optonets vs. day 4 for 2D cultures Shein et al., 2008). (2) The rate of these periodic synchronized bursts peaked during days 17–23 vs. day 21 in 2D cultures (Kamioka et al., 1996). (3) The so-called characteristic minute-to-minute fluctuations in synchronized burst rates (Habets et al., 1987) were manifested here also as “superbursts”—a bursting period extending over a few minutes, where burst frequency increased and was followed by a pause. Superbursts first appeared during days 10–16, but peaked between days 17–23. Similarly, minute-to-minute burst frequency fluctuations were present in 2D cultures with pronounced intensity on days 22–33 as compared to younger cultures (Habets et al., 1987), and declined with further maturation of the network. Apart from these similarities, there also appeared to be considerable differences between the two culture types. While 2D cultures present pronounced synchronized bursting, the maturation of 3D optonets was accompanied by the development of sporadic activity that became increasingly complex, with parallel development of synchronous activity that peaked at days 17–23 (Figure 5). Following this period, bursting activity declined and its frequency became less regular. The overall complexity of the 3D cultures developed over time, as opposed to the “mature state” that characterizes the synchronous bursting of 2D cultures.

A closer look at the cultures' calcium traces revealed that in the early stages *in vitro*, both sporadic and synchronized network events tended to exhibit rapid rise and decay dynamics, while mature cultures exhibited significantly longer durations for both sporadic and synchronized events (Figure 6). These characteristically slower dynamics could have multiple underlying causes. First, higher concentrations of a calcium indicator can lead to more prolonged (and relatively smaller) signals as a consequence of buffering (Helmchen et al., 1996). Although we carefully calibrated the viral vector titer to achieve consistent expression levels, we cannot rule out the possibility that increasing probe expression may have been a contributing factor. This effect, however, cannot explain the ultra-long burst durations seen for example, on day 23. These more likely reflect periods of high-frequency stereotyped bursting (Calvin and Loeser, 1975; Ogata, 1976) underlying the observed “super-bursts,” which are putatively too frequent to resolve given the poor temporal resolution of the indicator (which is further significantly lower during multi-spike events Chen et al., 2013). A final intriguing possibility which currently cannot be excluded, is that these prolonged spontaneous events could include regenerative calcium events or calcium waves.

In our previous studies, optonets were subjected to varying environmental conditions (see Dana et al., 2014; Marom, 2014; Marom et al., 2015 for the effects of different scaffold materials, support cells and pharmacological environments). Here, temperature was found to be a powerful modulator of spontaneous activity patterns; when reduced, wave patterns with diverse spatial characteristics were observed. Similar effects were previously observed under cholinergic pharmacological modulation (Marom et al., 2015), which induced propagation of linear and circular waves at velocities up to 2 μm/s. Here, when lowering the temperature, waves propagated at similar velocities (periods of 2–6 s of waves were seen in culture, see Figure 7B). These types of highly complex sporadic patterns are clearly a desirable property in a network activity model aimed at mimicking the developing and mature CNS. Such major differences in activity patterns were previously seen to result from profound perturbations, such as changing the source of the cultured neurons (Napoli and Obeid, 2016), and being able to obtain them in optonets using relatively minor perturbations appears to be a promising feature that should be further characterized in future studies.

In conclusion, this study further establishes Optonets as a relevant model system for capturing complex spontaneous activity patterns in an accessible 3D neural network. This model system allows for flexible tweaking of multiple parameters that directly influence the (observable) neuronal activity, and features activity patterns that systematically change as the optonet matures. The observed patterns are qualitatively similar to those observed in 2D networks, mimicking features that are observed in the developing brain *in-vivo*. Moreover, the fact that its activity is qualitatively more complex and can be further desynchronized by varying the temperature, suggests a major relative advantage over 2D systems, since the synchronously-locked spontaneous bursts exhibited by 2D cultures limit their utility as a tool for studying various aspects of cellular physiology such as activity-dependent synaptic plasticity. Overall, our results suggest that combining a 3D optonet model with fast, high spatiotemporal resolution network activity probing provides a potent *in vitro* research tool.

## Ethics statement

The animal experiments and procedures were approved by the Institutional Animal Care Committee at Technion – Israel Institute of Technology and were in accordance with the National Institutes of Health Guide for the Care and Use of Laboratory Animals.

## Author contributions

AM, ES contributed equally to this work. SS, AM, and ES designed the study. ES developed the activity image processing, and AM developed the 3D network growth protocol. GCaMP6 transfection was performed by AM Stainings and confocal imaging were performed by AM Imaging was performed by AM and ES. SS and SL supervised the project. The manuscript was prepared jointly by all authors.

## Funding

This research was supported by the European Research Council (ERC) under the European Union's Horizon 2020 research and innovation program, # 641171.

### Conflict of interest statement

The authors declare that the research was conducted in the absence of any commercial or financial relationships that could be construed as a potential conflict of interest.
